# Supplementary material for: High resolution discovery of chromatin interactions
Source: Nucleic Acids Res. 2019 Feb 14;47(6):e35. doi: 10.1093/nar/gkz051 (PMC6451139; doi:10.1093/nar/gkz051)
Supplement: Supplementary Data [file gkz051_supplemental_file.pdf]

Supplemental Data

High resolution discovery of chromatin interactions

Yuchun Guo[1], Konstantin Krismer[1,2], Michael Closser[3], Hynek Wichterle[3], David K Gifford[1,2,*]

Keywords: Chromatin interaction; ChIA-PET; HiChIP; Chromatin loop

[1]Computer Science and Artificial Intelligence Laboratory, Massachusetts Institute of Technology, Cambridge, MA

[2]Department of Biological Engineering, Massachusetts Institute of Technology, Cambridge, MA

[3]Departments of Pathology and Cell Biology, Neurology, and Neuroscience, Center for Motor Neuron Biology and Disease, Columbia University Medical Center, New York, NY

* Corresponding author, gifford@mit.edu

Table S1. ChIA-PET and HiChIP datasets used in this study

Dataset identifier	Target	Cell line	ENCODE/GEO
Ruan.K562.POLR2A	POLR2A	K562	ENCSR000BZY
Ruan.MCF-7.POLR2A	POLR2A	MCF-7	ENCSR000CAA
Snyder.K562.POLR2A	POLR2A	K562	ENCSR000FDC
Ruan.MCF-7.CTCF	CTCF	MCF-7	ENCSR000CAD
Snyder.GM12878.RAD21.2014	RAD21	GM12878	ENCSR752QCX
Snyder.GM12878.RAD21.2016	RAD21	GM12878	ENCSR981FNA
Snyder.GM12891.RAD21	RAD21	GM12891	ENCSR299VMZ
Snyder.GM12892.RAD21	RAD21	GM12892	ENCSR033GUP
Snyder.GM19238.RAD21	RAD21	GM19238	ENCSR527RXH
Snyder.GM19239.RAD21	RAD21	GM19239	ENCSR479MTN
Snyder.GM19240.RAD21	RAD21	GM19240	ENCSR312TUD
Snyder.HepG2.RAD21	RAD21	HepG2	ENCSR014ZXR
Snyder.JurkatCloneE61.RAD21	RAD21	Jurkat clone E61	ENCSR465NNU
Snyder.K562.RAD21	RAD21	K562	ENCSR000FDB
Snyder.LNCaPCloneFGC.RAD21	RAD21	LNCaP clone FGC	ENCSR011ITK
Snyder.MCF-7.RAD21	RAD21	MCF-7	ENCSR716WZI
Snyder.SU-DHL-2.RAD21	RAD21	SU-DHL-2	ENCSR466AXT
Chang.GM12878.SMC1A.HiChIP	SMC1A	GM12878	GSE80820

data set	method	replicate 1		replicate 2		common		Jaccard coefficient	
		min	full	min	full	min	full	min	full
Ruan.K562.POLR2A	ChIA-PET2	613	17,608	630	18,575	397	10,520	0.47	0.41
	CID	613	129,977	630	137,239	499	74,326	0.67	0.39
	Mango	613	613	630	630	317	317	0.34	0.34
Ruan.MCF-7.CTCF	ChIA-PET2	13,076	32,530	4239	10,168	2922	6542	0.2	0.18
	CID	13076	68,498	4239	38,543	3856	21,200	0.29	0.25
	Mango	13076	13,076	4239	4239	3072	3072	0.22	0.22
Ruan.MCF-7.POLR2A	ChIA-PET2	1417	18,574	2392	35,525	704	9059	0.23	0.2
	CID	1417	115,124	2392	171,345	1251	71,170	0.49	0.33
	Mango	1417	1417	2392	2392	782	782	0.26	0.26
Snyder.GM12878.RAD21.2014	ChIA-PET2	5657	25,683	4769	15,451	3417	8873	0.49	0.28
	CID	5657	61,966	4769	46,076	3589	22,348	0.52	0.26
	Mango	5657	5657	4769	4769	2573	2573	0.33	0.33
Snyder.GM12878.RAD21.2016	ChIA-PET2	28,436	347,013	18,469	211,124	15,315	110,031	0.48	0.25
	CID	28,436	360,920	18,469	227,912	16,865	141,545	0.56	0.32
	Mango	28,436	28,436	18,469	18,469	13,896	13,896	0.42	0.42
Snyder.GM12891.RAD21	ChIA-PET2	5806	28,200	18,000	63,328	5427	16,234	0.3	0.22
	CID	5806	58,503	18,000	176,938	5575	40,168	0.31	0.21
	Mango	5806	5806	18,000	18,000	4802	4802	0.25	0.25
Snyder.GM12892.RAD21	ChIA-PET2	7721	34,168	6841	22,766	4691	12,775	0.48	0.29
	CID	7721	87,567	6841	89,597	5228	36,901	0.56	0.26
	Mango	7721	7721	6841	6841	4024	4024	0.38	0.38
Snyder.GM19238.RAD21	ChIA-PET2	16,791	67,480	2150	16,697	2020	13,725	0.12	0.19
	CID	16,791	186,952	2150	49,182	2140	32,278	0.13	0.16
	Mango	16,791	16,791	2150	2150	1865	1865	0.11	0.11
Snyder.GM19239.RAD21	ChIA-PET2	9763	38,075	96	1233	91	1088	0.01	0.03
	CID	9763	120,478	96	8528	96	6384	0.01	0.05
	Mango	9763	9763	96	96	84	84	0.01	0.01
Snyder.GM19240.RAD21	ChIA-PET2	1354	3925	514	7782	321	1953	0.21	0.2
	CID	1354	21,200	514	16,699	404	6201	0.28	0.2
	Mango	1354	1354	514	514	180	180	0.11	0.11
Snyder.HepG2.RAD21	ChIA-PET2	4757	47,028	5444	50,259	2987	20,986	0.41	0.28
	CID	4757	56,009	5444	54,724	3279	22,151	0.47	0.25
	Mango	4757	4757	5444	5444	2247	2247	0.28	0.28
Snyder.JurkatCloneE61.RAD21	ChIA-PET2	3603	22,426	3932	25,379	2444	11,188	0.48	0.31
	CID	3603	53,831	3932	58,485	2633	19,831	0.54	0.21
	Mango	3603	3603	3932	3932	1533	1533	0.26	0.26
Snyder.K562.POLR2A	ChIA-PET2	1415	53,273	1093	17,408	139	7939	0.06	0.13
	CID	1415	70,337	1093	59,315	808	29,175	0.48	0.29
	Mango	1415	1415	1093	1093	472	472	0.23	0.23
Snyder.K562.RAD21	ChIA-PET2	1309	3951	5489	28,873	1140	2998	0.2	0.1
	CID	1309	19,100	5489	95,171	1226	12,686	0.22	0.12
	Mango	1309	1309	5489	5489	875	875	0.15	0.15
Snyder.LNCaPCloneFGC.RAD21	ChIA-PET2	4364	29,531	4618	28,990	2355	14,717	0.36	0.34
	CID	4364	81,198	4618	105,439	3111	28,028	0.53	0.18
	Mango	4364	4364	4618	4618	2071	2071	0.3	0.3
Snyder.MCF-7.RAD21	ChIA-PET2	3982	56,768	6969	38,262	1404	12,343	0.15	0.15
	CID	3982	46,099	6969	80,469	3127	23,336	0.4	0.23
	Mango	3982	3982	6969	6969	2365	2365	0.28	0.28
Snyder.SU-DHL-2.RAD21	ChIA-PET2	17,010	100,912	13,781	68,198	9860	38,662	0.47	0.3
	CID	17,010	123,855	13,781	96,652	11,230	55,738	0.57	0.34
	Mango	17,010	17,010	13,781	13,781	8742	8742	0.4	0.4

Table S2: Replicate consistency of ChIA-PET2, CID, and Mango interactions across 17 ENCODE data sets. Columns *replicate 1* and *replicate 2* contains interaction counts per method and data set for the respective replicates. Only interactions with $FDR < 0.05$ are counted. Column *common* contains the number of interactions present in both replicate 1 and 2 (i.e., replicated interactions). An interaction is considered replicated if there exists an interaction in the other replicate for which both anchors overlap with the original interaction (± 1000 bp). The last column contains the Jaccard coefficient of the sets of interactions in replicate 1 and 2. The *min* columns contain numbers based on the first n interactions, where n is determined by the method with the lowest number of significant interactions ($FDR < 0.05$). These interactions are used to compare replicate consistency across methods. The *full* columns include numbers based on all significant interactions for the respective replicates.

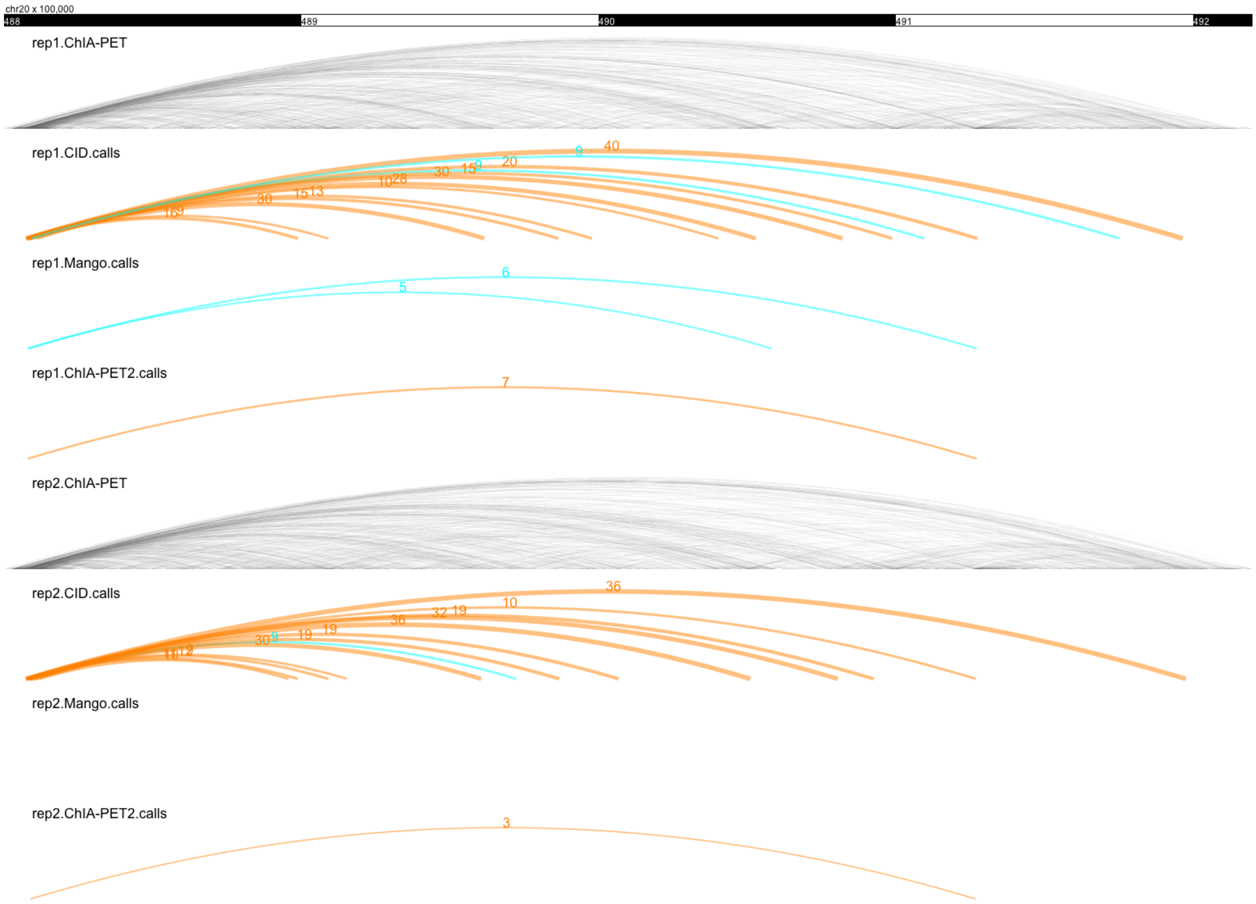

Figure S1. CID is more sensitive and consistent at discovering ChIA-PET interactions than peak-calling-based methods. Comparison of interactions called by CID, ChIA-PET2, and Mango in the CEBPB locus using two POLR2A ChIA-PET replicates from K562 cells. The PET counts of the interactions are represented as the numeric values above the arcs. Arcs in orange represent significant interactions that are replicable across biological replicates. Arcs in cyan represent significant interactions that are not replicable across biological replicates.

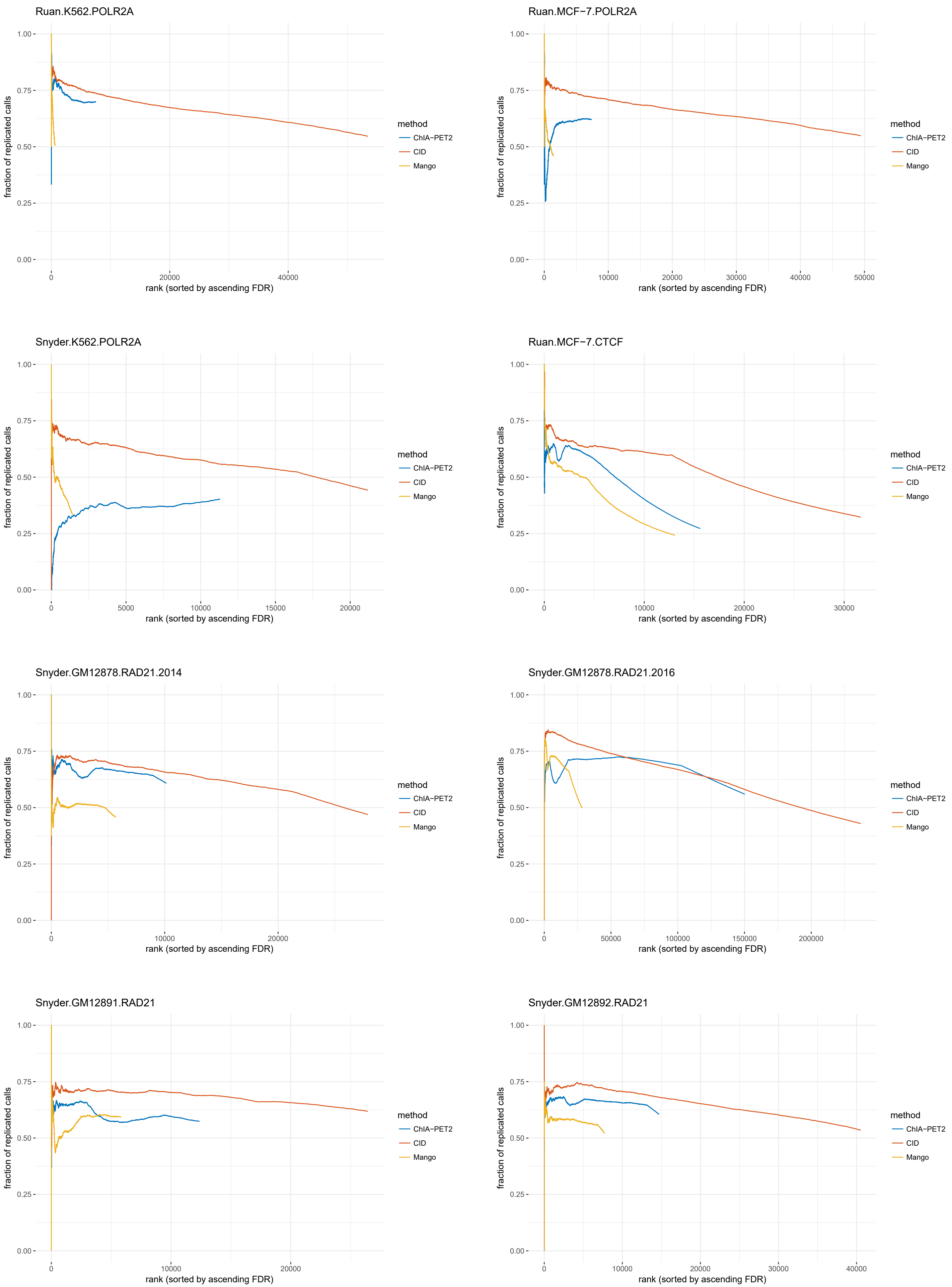
Ruan.K562.POLR2A
Ruan.MCF−7.POLR2A
Snyder.K562.POLR2A
Ruan.MCF−7.CTCF
Snyder.GM12878.RAD21.2014
Snyder.GM12878.RAD21.2016
Snyder.GM12891.RAD21
Snyder.GM12892.RAD21
fraction of replicated calls
0.00
0.25
0.50
0.75
1.00
rank (sorted by ascending FDR)
0
5000
10000
15000
20000
30000
40000
50000
100000
150000
200000
method
ChIA−PET2
CID
Mango

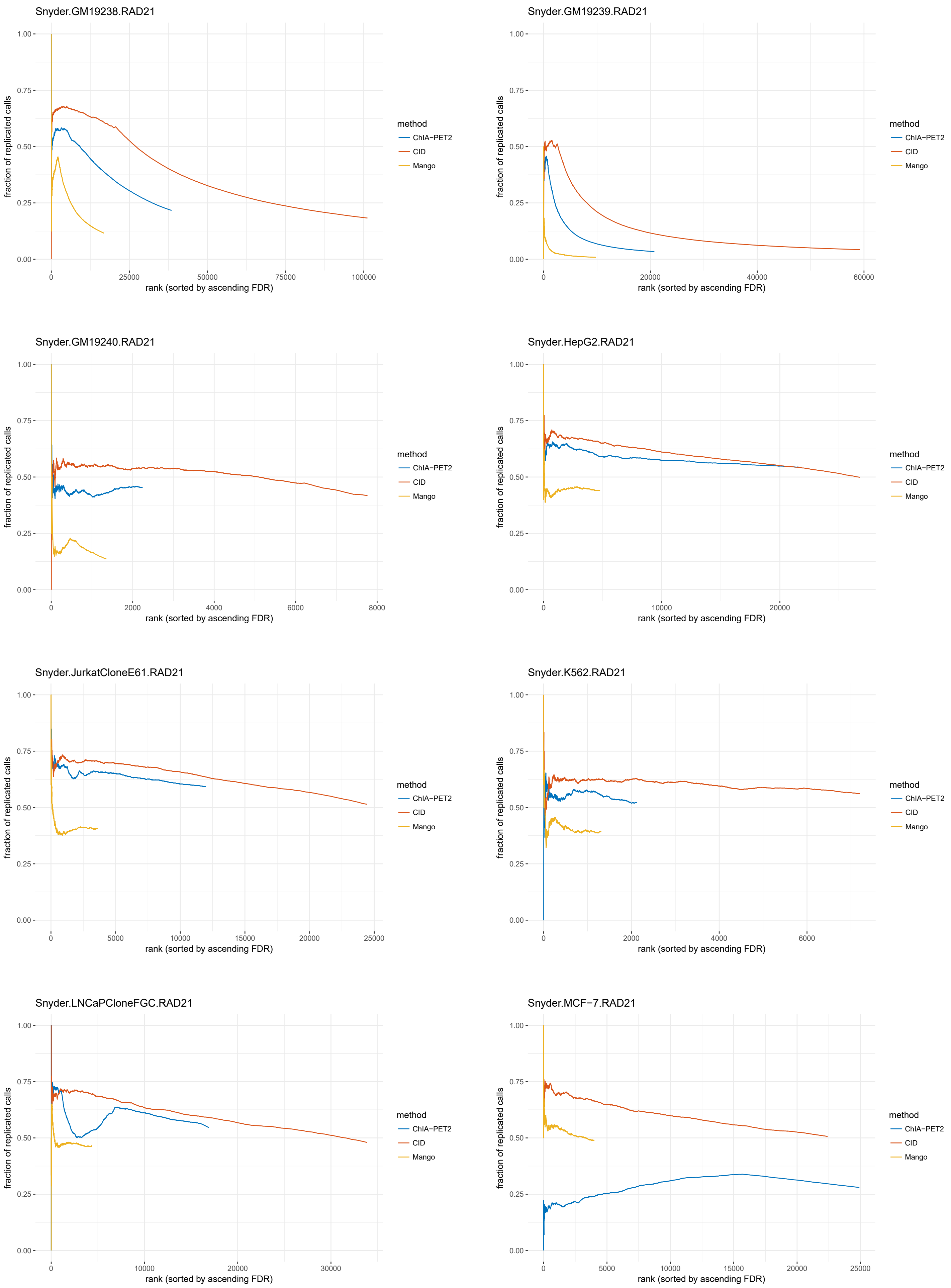
Snyder.GM19238.RAD21
fraction of replicated calls
1.00
0.75
0.50
0.25
0.00
0
25000
50000
75000
100000
rank (sorted by ascending FDR)
method
ChIA−PET2
CID
Mango
Snyder.GM19239.RAD21
fraction of replicated calls
1.00
0.75
0.50
0.25
0.00
0
20000
40000
60000
rank (sorted by ascending FDR)
method
ChIA−PET2
CID
Mango
Snyder.GM19240.RAD21
fraction of replicated calls
1.00
0.75
0.50
0.25
0.00
0
2000
4000
6000
8000
rank (sorted by ascending FDR)
method
ChIA−PET2
CID
Mango
Snyder.HepG2.RAD21
fraction of replicated calls
1.00
0.75
0.50
0.25
0.00
0
10000
20000
rank (sorted by ascending FDR)
method
ChIA−PET2
CID
Mango
Snyder.JurkatCloneE61.RAD21
fraction of replicated calls
1.00
0.75
0.50
0.25
0.00
0
5000
10000
15000
20000
25000
rank (sorted by ascending FDR)
method
ChIA−PET2
CID
Mango
Snyder.K562.RAD21
fraction of replicated calls
1.00
0.75
0.50
0.25
0.00
0
2000
4000
6000
rank (sorted by ascending FDR)
method
ChIA−PET2
CID
Mango
Snyder.LNCaPCloneFGC.RAD21
fraction of replicated calls
1.00
0.75
0.50
0.25
0.00
0
10000
20000
30000
rank (sorted by ascending FDR)
method
ChIA−PET2
CID
Mango
Snyder.MCF−7.RAD21
fraction of replicated calls
1.00
0.75
0.50
0.25
0.00
0
5000
10000
15000
20000
25000
rank (sorted by ascending FDR)
method
ChIA−PET2
CID
Mango

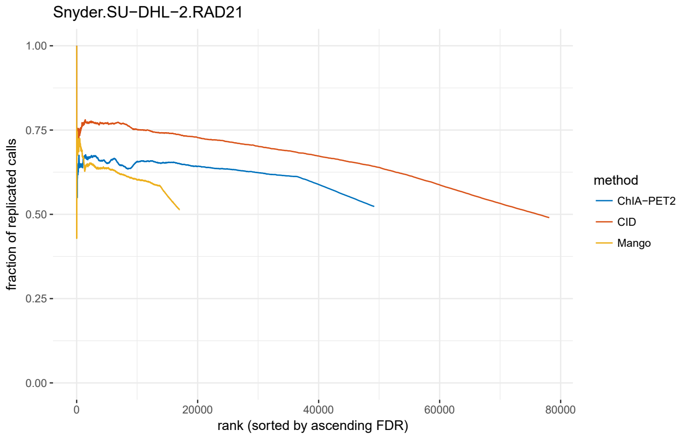

Figure S2. Interaction calls of CID are more consistent across replicates than those of ChIA-PET2 and Mango. Accumulative fractions of replicated interaction calls are computed using top ranking interactions at increasing ranks.

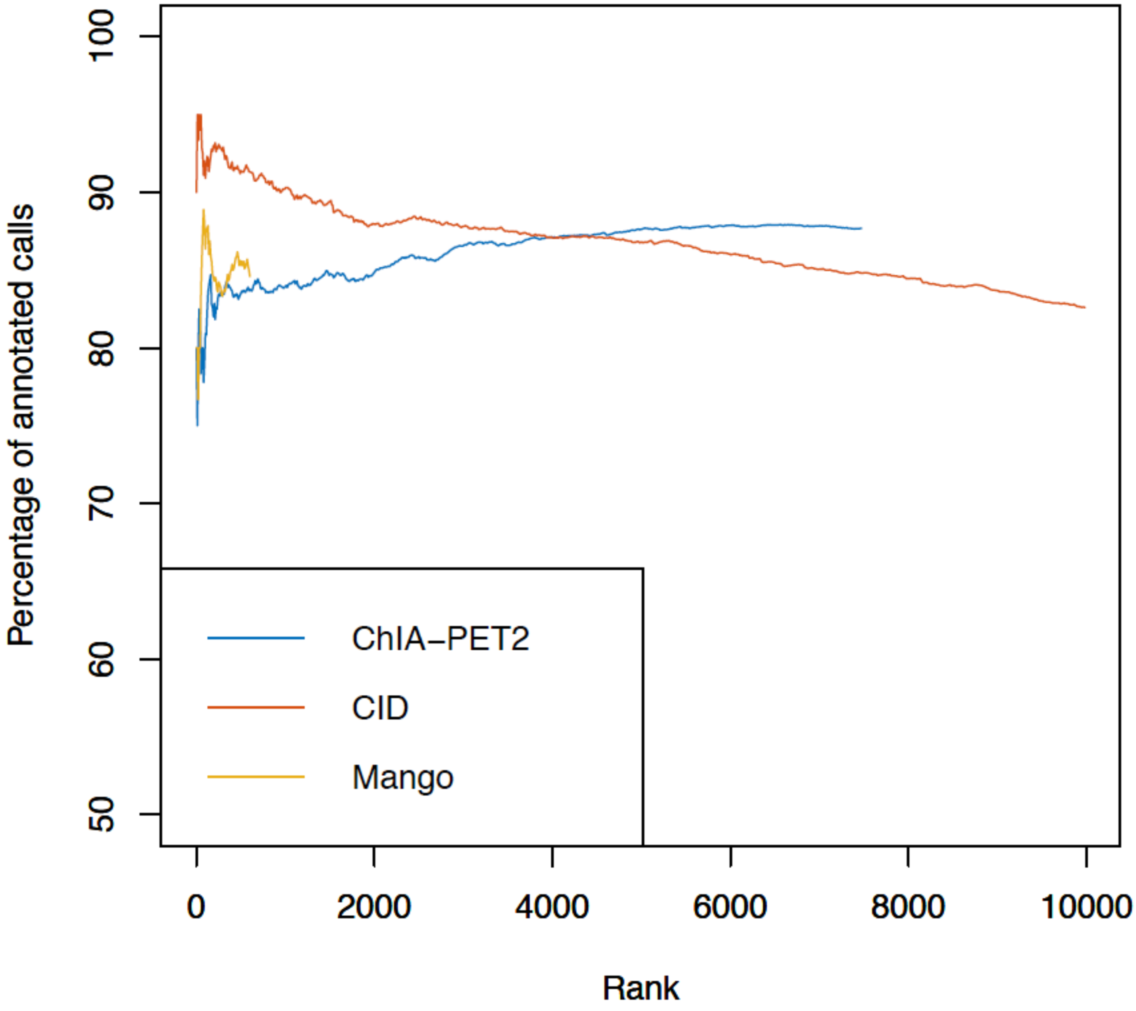

Figure S3. The accumulative percentage of POL2RA ChIA-PET interaction calls from three methods that are annotated as enhancer-promoter interactions. An interaction call is annotated as enhancer-promoter interaction if one of its anchor regions overlaps with at least one promoter or enhancer annotation and the other anchor region overlaps with at least one annotation of the opposite type (E-P or P-E). The accumulative percentage values are computed using top ranking interactions at increasing ranks.

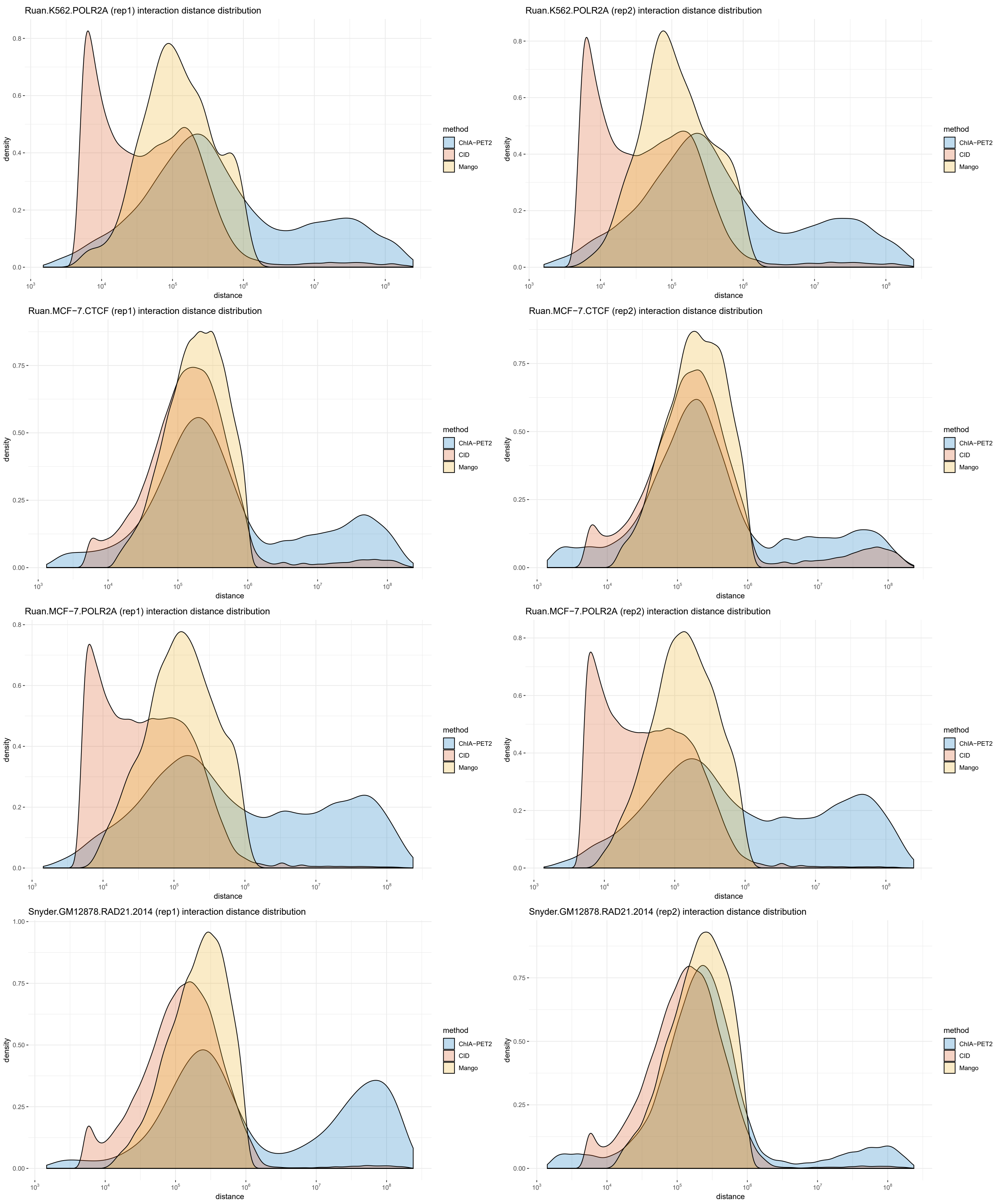
Ruan.K562.POLR2A (rep1) interaction distance distribution
Ruan.K562.POLR2A (rep2) interaction distance distribution
Ruan.MCF-7.CTCF (rep1) interaction distance distribution
Ruan.MCF-7.CTCF (rep2) interaction distance distribution
Ruan.MCF-7.POLR2A (rep1) interaction distance distribution
Ruan.MCF-7.POLR2A (rep2) interaction distance distribution
Snyder.GM12878.RAD21.2014 (rep1) interaction distance distribution
Snyder.GM12878.RAD21.2014 (rep2) interaction distance distribution
density
distance
method
ChIA-PET2
CID
Mango

Snyder.GM12878.RAD21.2016 (rep1) interaction distance distribution

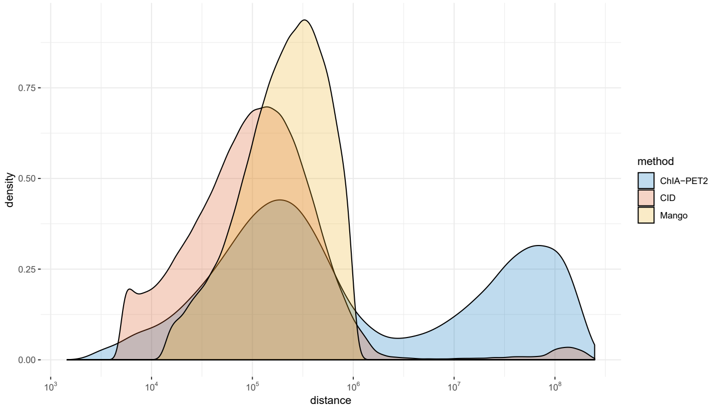

Snyder.GM12878.RAD21.2016 (rep2) interaction distance distribution

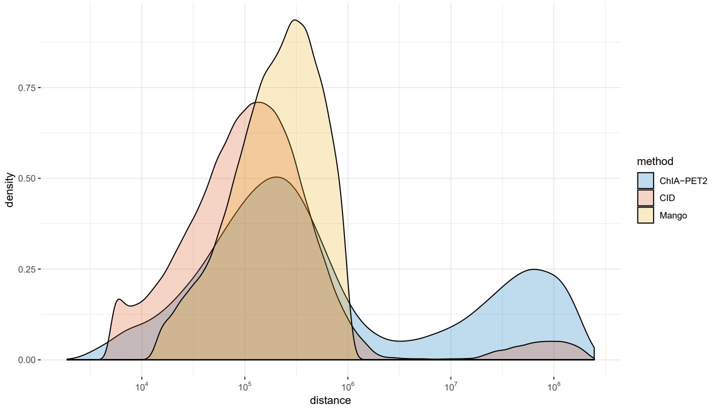

Snyder.GM12891.RAD21 (rep1) interaction distance distribution

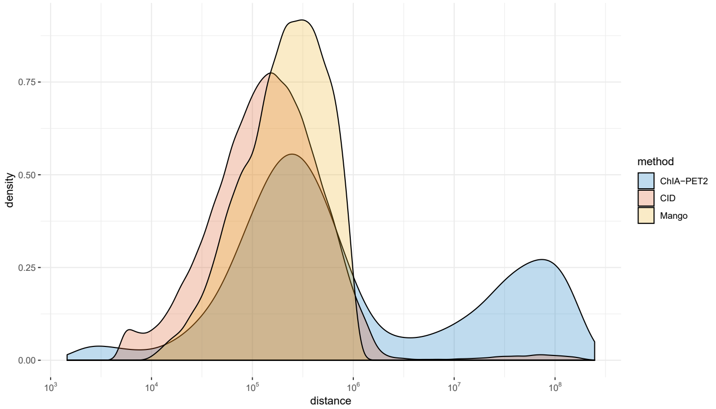

Snyder.GM12891.RAD21 (rep2) interaction distance distribution

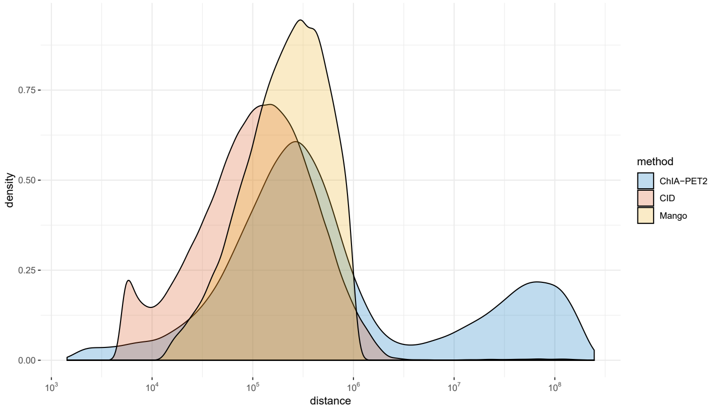

Snyder.GM12892.RAD21 (rep1) interaction distance distribution

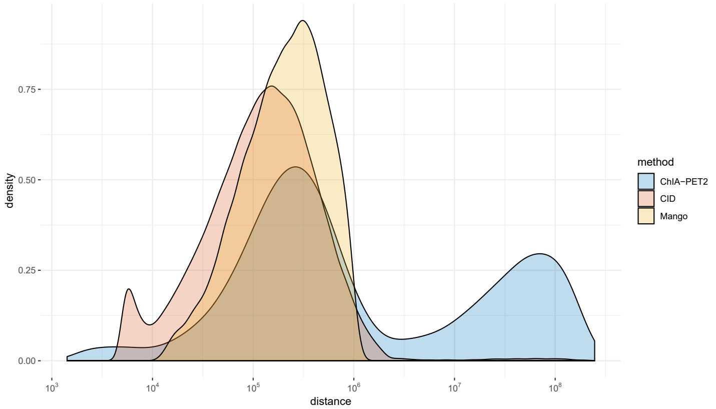

Snyder.GM12892.RAD21 (rep2) interaction distance distribution

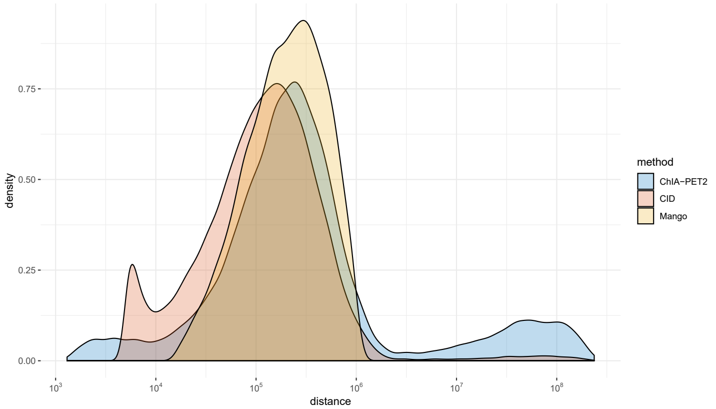

Snyder.GM19238.RAD21 (rep1) interaction distance distribution

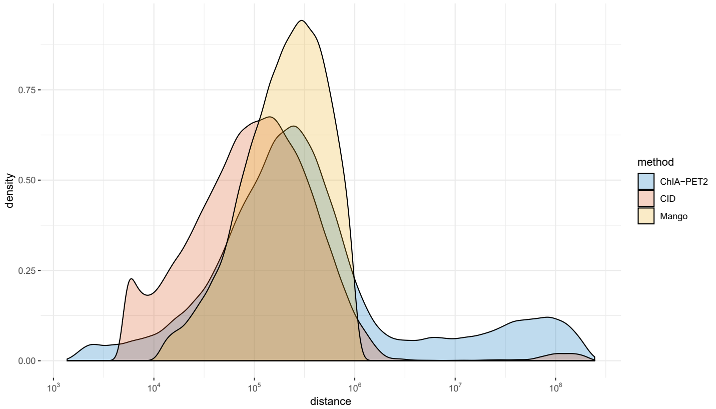

Snyder.GM19238.RAD21 (rep2) interaction distance distribution

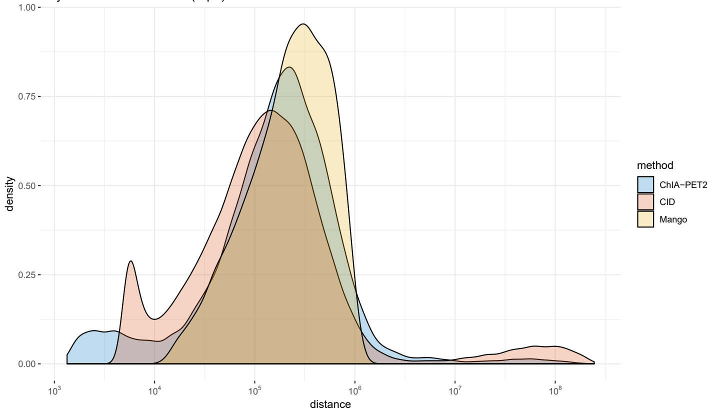

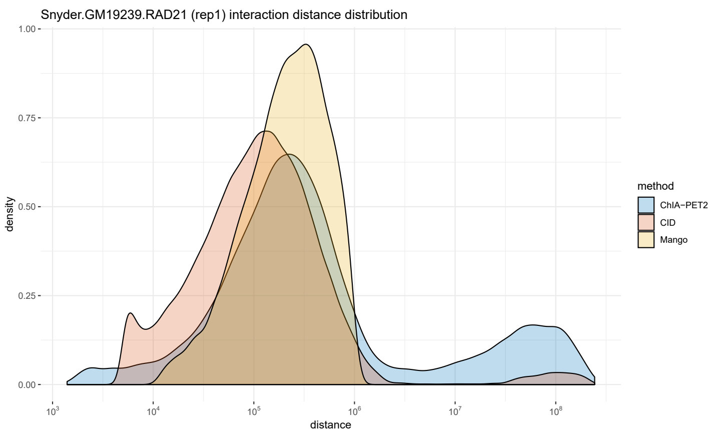
Snyder.GM19239.RAD21 (rep1) interaction distance distribution
density
1.00
0.75
0.50
0.25
0.00
distance
method
ChIA−PET2
CID
Mango

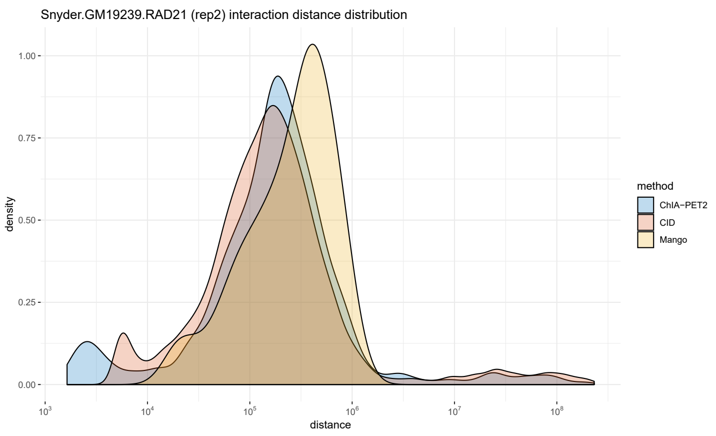
Snyder.GM19239.RAD21 (rep2) interaction distance distribution
density
1.00
0.75
0.50
0.25
0.00
distance
method
ChIA−PET2
CID
Mango

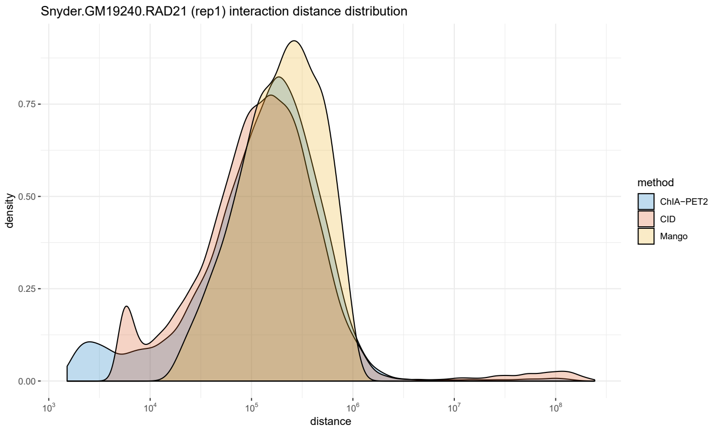
Snyder.GM19240.RAD21 (rep1) interaction distance distribution
density
0.75
0.50
0.25
0.00
distance
method
ChIA−PET2
CID
Mango

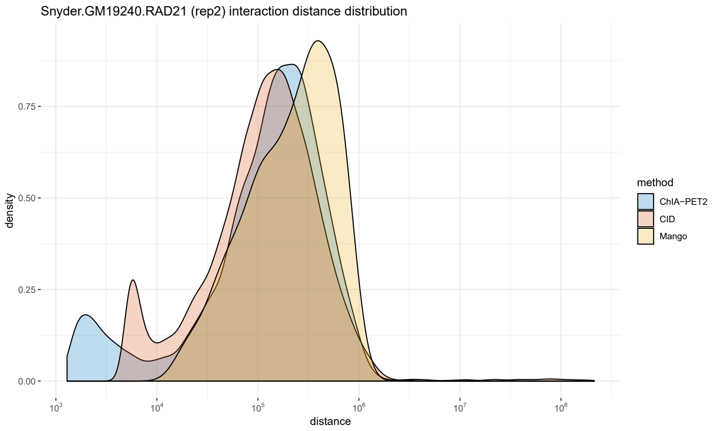
Snyder.GM19240.RAD21 (rep2) interaction distance distribution
density
0.75
0.50
0.25
0.00
distance
method
ChIA−PET2
CID
Mango

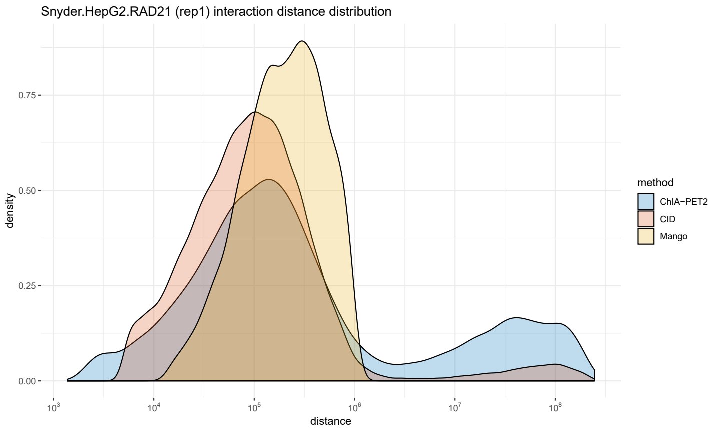
Snyder.HepG2.RAD21 (rep1) interaction distance distribution
density
0.75
0.50
0.25
0.00
distance
method
ChIA−PET2
CID
Mango

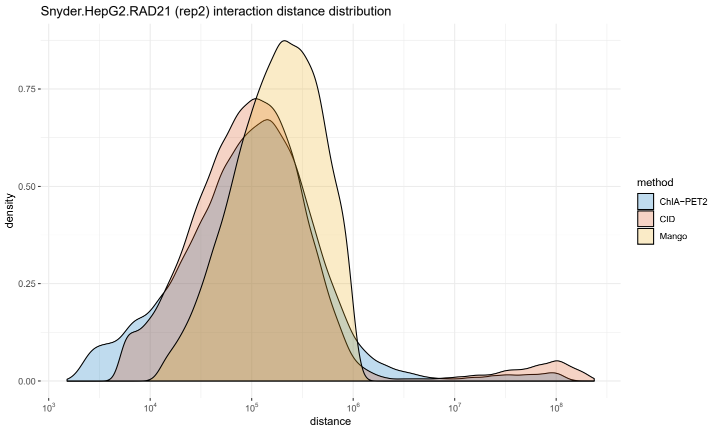
Snyder.HepG2.RAD21 (rep2) interaction distance distribution
density
0.75
0.50
0.25
0.00
distance
method
ChIA−PET2
CID
Mango

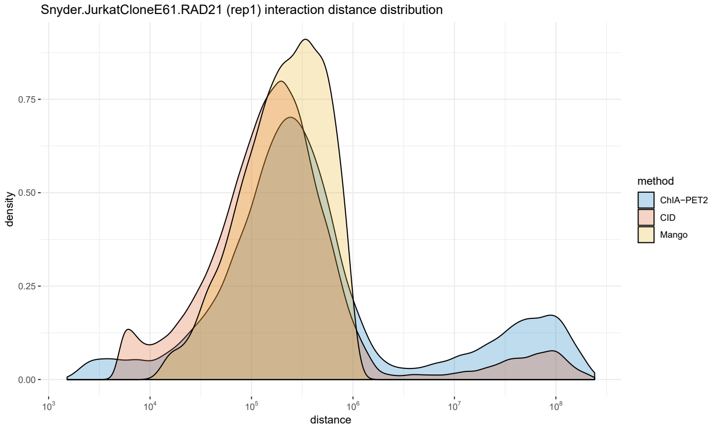
Snyder.JurkatCloneE61.RAD21 (rep1) interaction distance distribution
density
0.75
0.50
0.25
0.00
distance
method
ChIA−PET2
CID
Mango

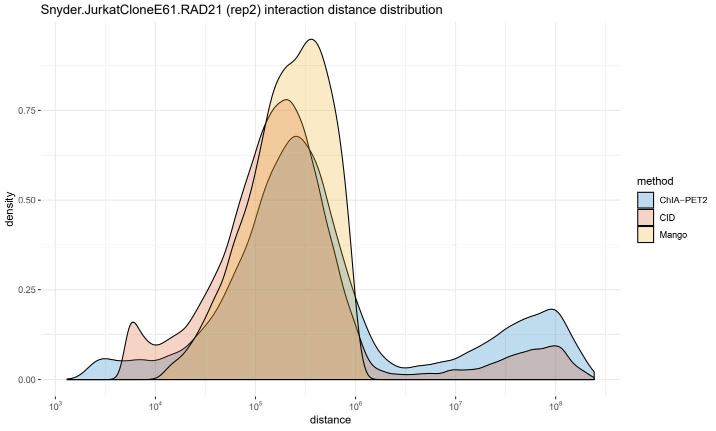
Snyder.JurkatCloneE61.RAD21 (rep2) interaction distance distribution
density
0.75
0.50
0.25
0.00
distance
method
ChIA−PET2
CID
Mango

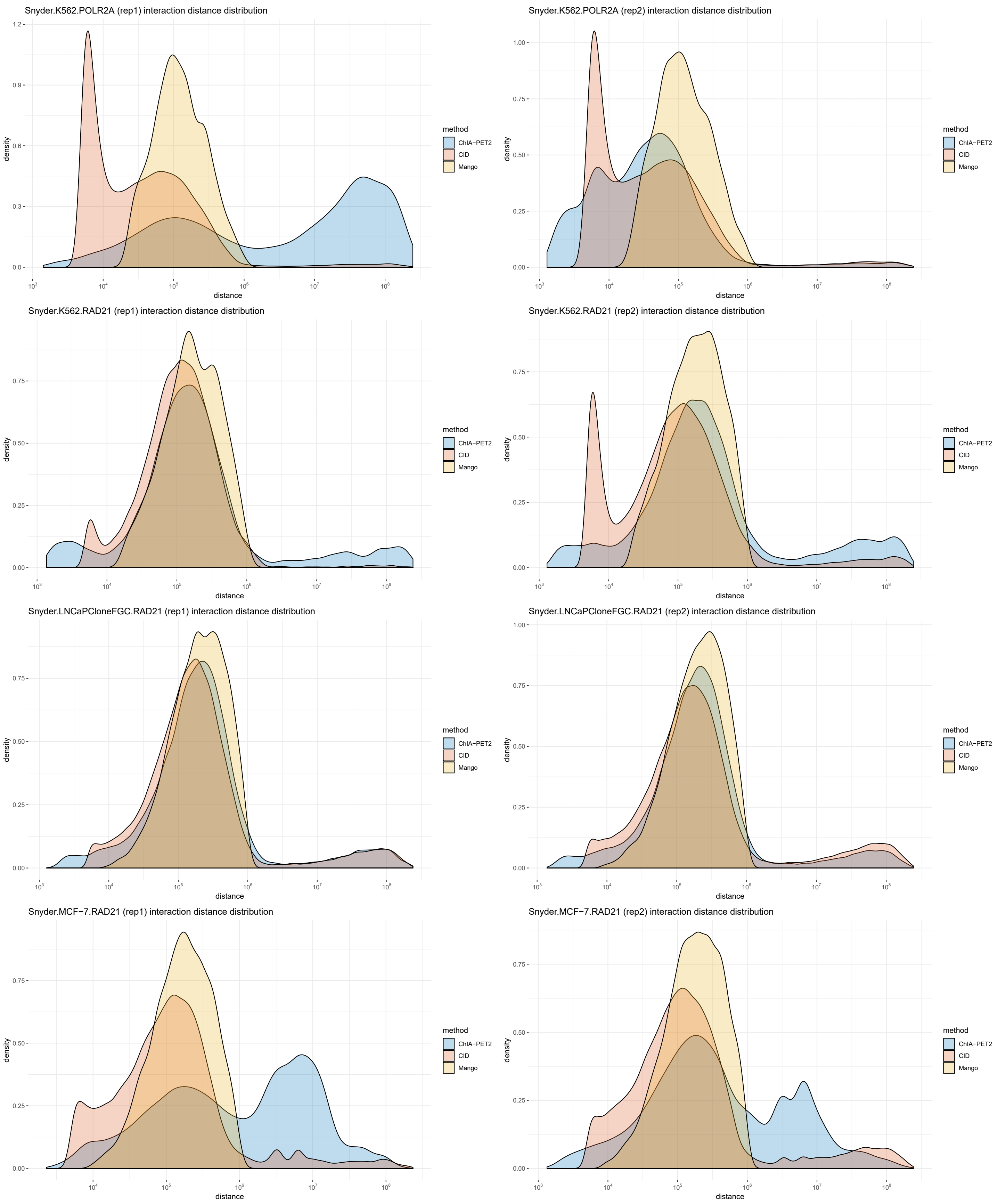
Snyder.K562.POLR2A (rep1) interaction distance distribution
Snyder.K562.POLR2A (rep2) interaction distance distribution
Snyder.K562.RAD21 (rep1) interaction distance distribution
Snyder.K562.RAD21 (rep2) interaction distance distribution
Snyder.LNCaPCloneFGC.RAD21 (rep1) interaction distance distribution
Snyder.LNCaPCloneFGC.RAD21 (rep2) interaction distance distribution
Snyder.MCF−7.RAD21 (rep1) interaction distance distribution
Snyder.MCF−7.RAD21 (rep2) interaction distance distribution
density
distance
method
ChIA−PET2
CID
Mango

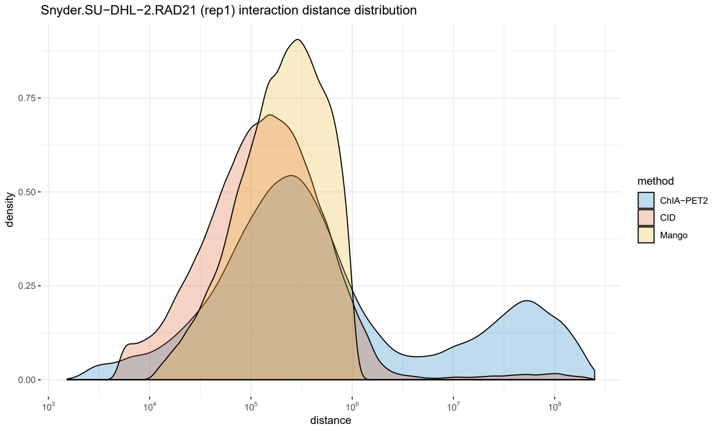

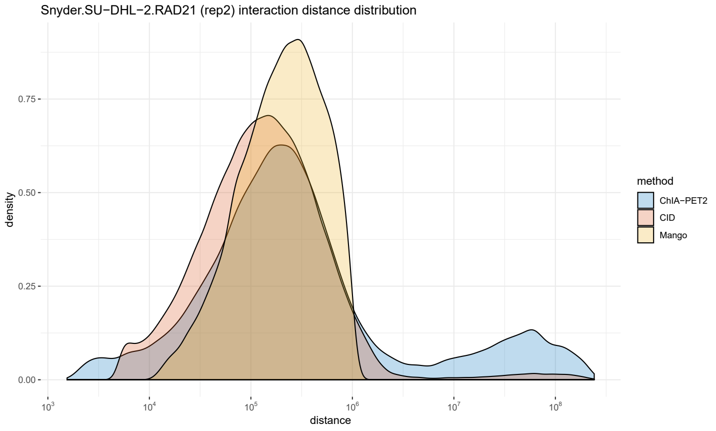

Figure S4: Comparing interaction length distributions of significant interactions identified by ChIA-PET2, CID, and Mango across 17 ENCODE data sets. The length of an interaction is determined by the distance between its anchor regions. Only intra-chromosomal interactions are considered.

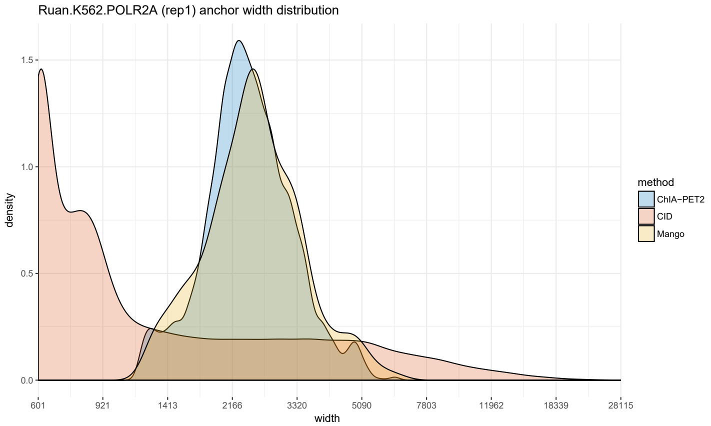
Ruan.K562.POLR2A (rep1) anchor width distribution
density
0.0
0.5
1.0
1.5
601
921
1413
2166
3320
5090
7803
11962
18339
28115
width
method
ChIA−PET2
CID
Mango

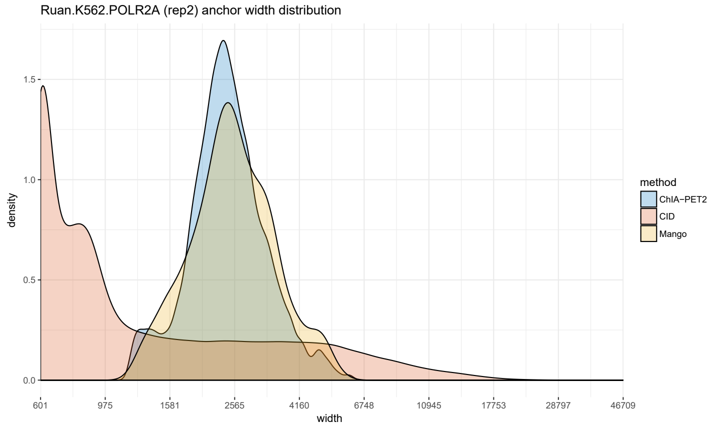
Ruan.K562.POLR2A (rep2) anchor width distribution
density
0.0
0.5
1.0
1.5
601
975
1581
2565
4160
6748
10945
17753
28797
46709
width
method
ChIA−PET2
CID
Mango

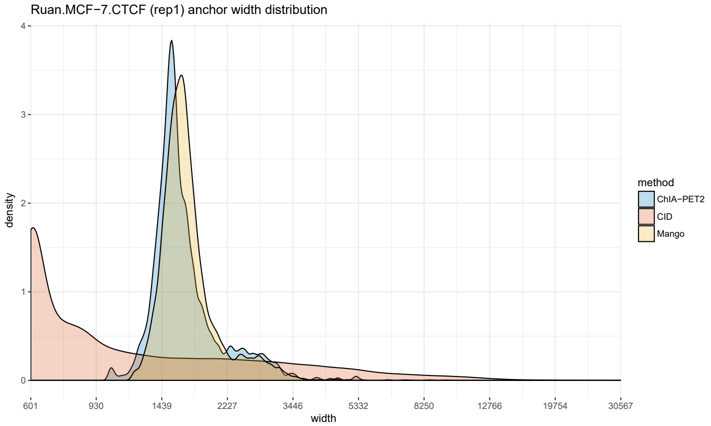
Ruan.MCF−7.CTCF (rep1) anchor width distribution
density
0
1
2
3
4
601
930
1439
2227
3446
5332
8250
12766
19754
30567
width
method
ChIA−PET2
CID
Mango

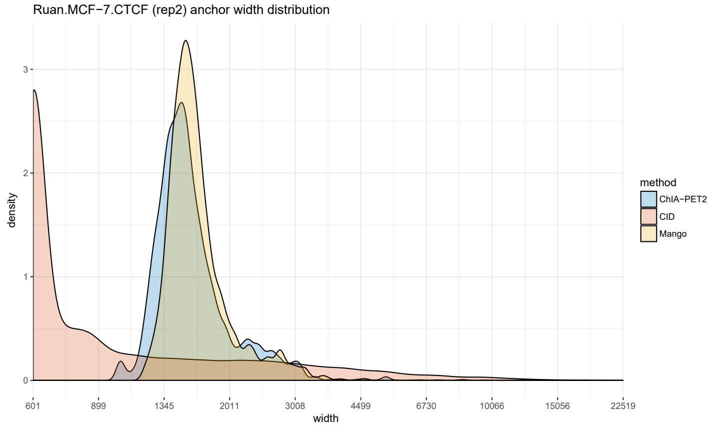
Ruan.MCF−7.CTCF (rep2) anchor width distribution
density
0
1
2
3
601
899
1345
2011
3008
4499
6730
10066
15056
22519
width
method
ChIA−PET2
CID
Mango

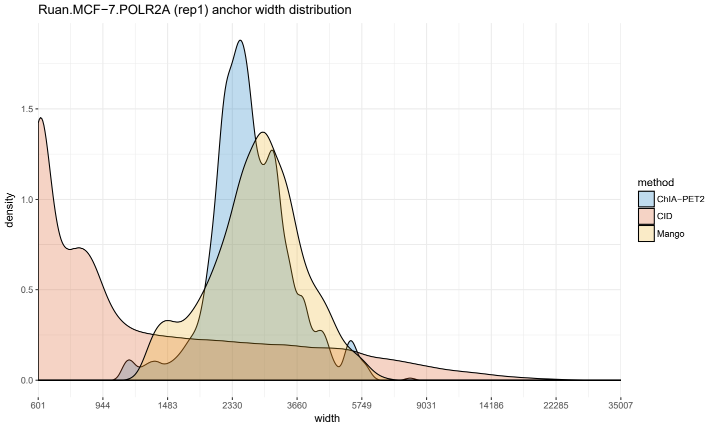
Ruan.MCF−7.POLR2A (rep1) anchor width distribution
density
0.0
0.5
1.0
1.5
601
944
1483
2330
3660
5749
9031
14186
22285
35007
width
method
ChIA−PET2
CID
Mango

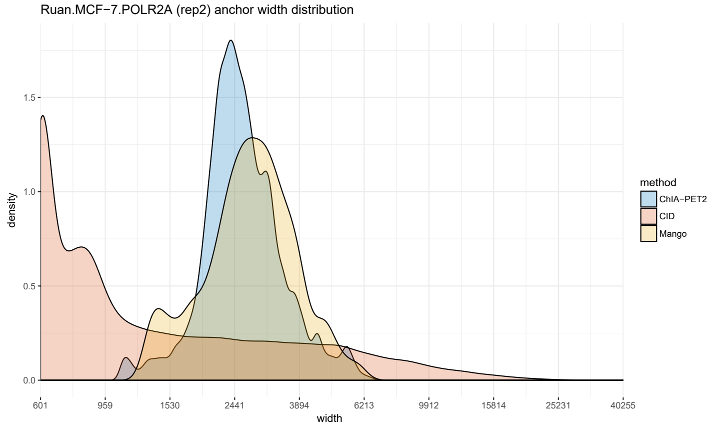
Ruan.MCF−7.POLR2A (rep2) anchor width distribution
density
0.0
0.5
1.0
1.5
601
959
1530
2441
3894
6213
9912
15814
25231
40255
width
method
ChIA−PET2
CID
Mango

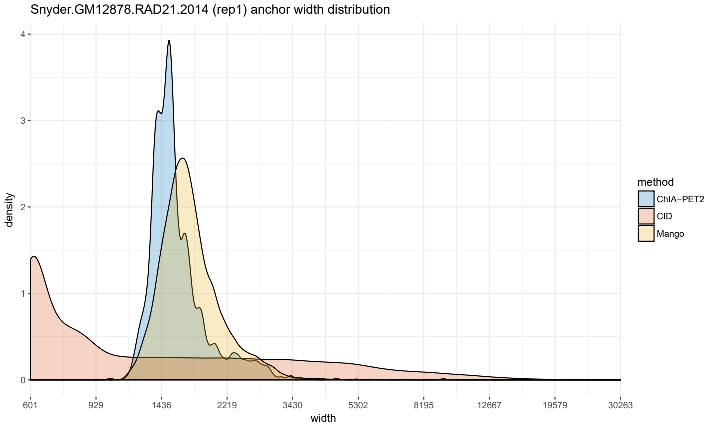
Snyder.GM12878.RAD21.2014 (rep1) anchor width distribution
density
0
1
2
3
4
601
929
1436
2219
3430
5302
8195
12667
19579
30263
width
method
ChIA−PET2
CID
Mango

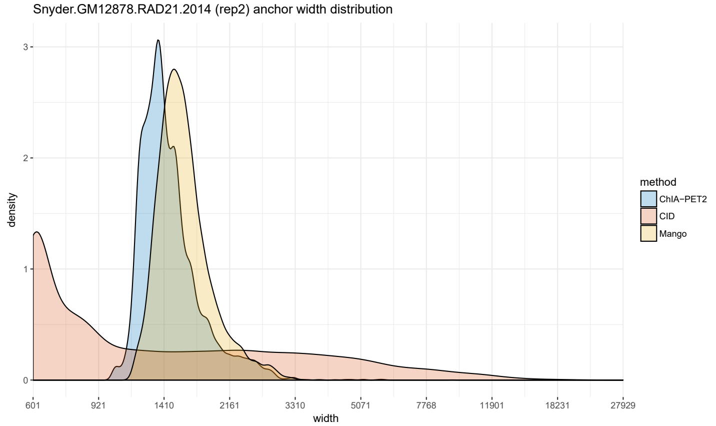
Snyder.GM12878.RAD21.2014 (rep2) anchor width distribution
density
0
1
2
3
601
921
1410
2161
3310
5071
7768
11901
18231
27929
width
method
ChIA−PET2
CID
Mango

Snyder.GM12878.RAD21.2016 (rep1) anchor width distribution

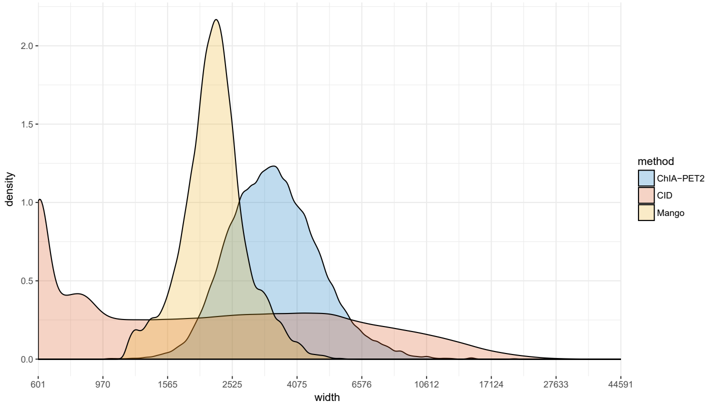

Snyder.GM12878.RAD21.2016 (rep2) anchor width distribution

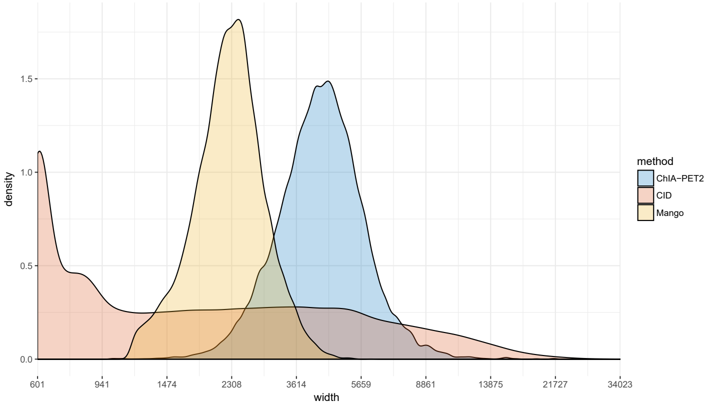

Snyder.GM12891.RAD21 (rep1) anchor width distribution

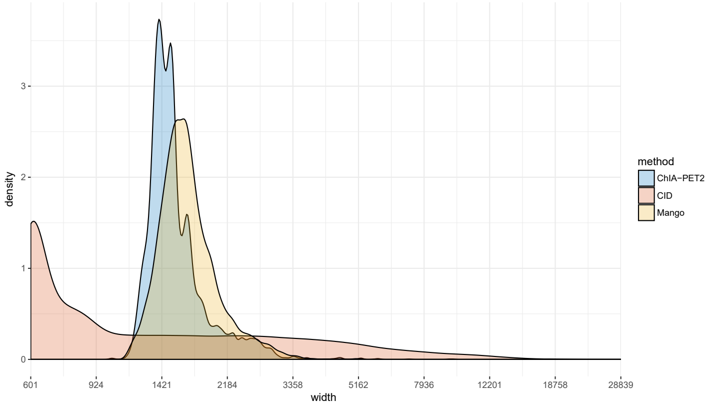

Snyder.GM12891.RAD21 (rep2) anchor width distribution

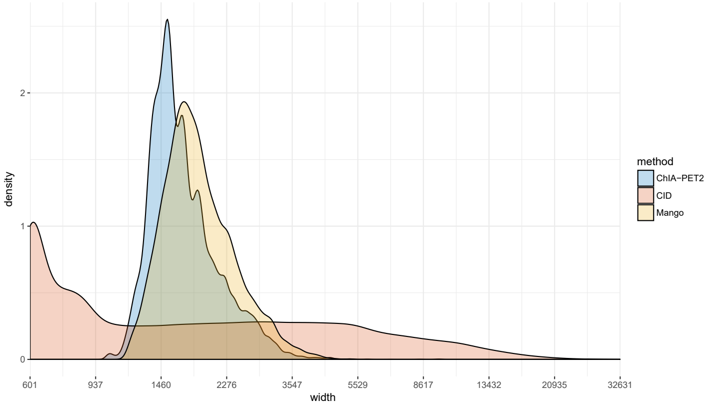

Snyder.GM12892.RAD21 (rep1) anchor width distribution

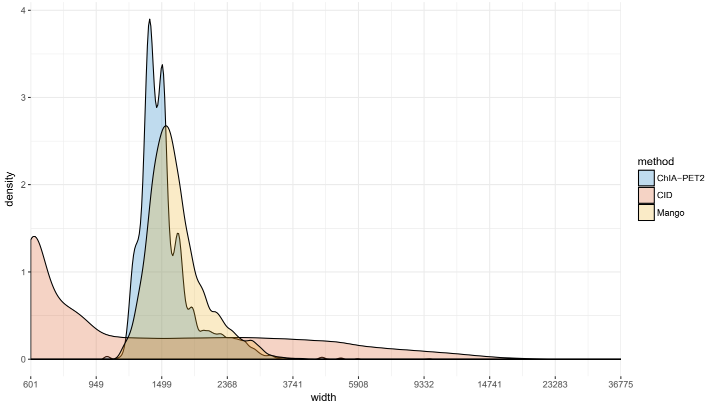

Snyder.GM12892.RAD21 (rep2) anchor width distribution

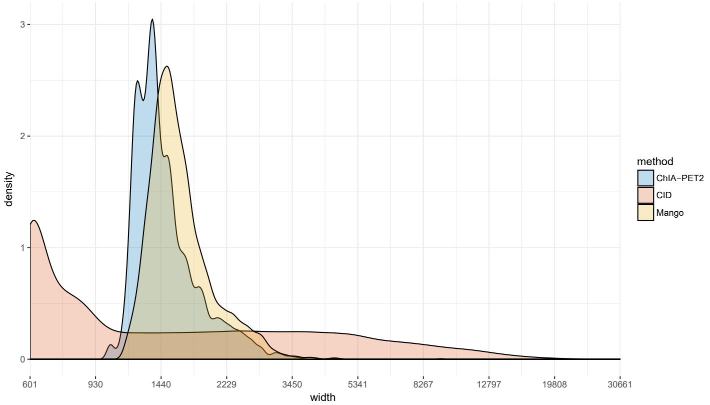

Snyder.GM19238.RAD21 (rep1) anchor width distribution

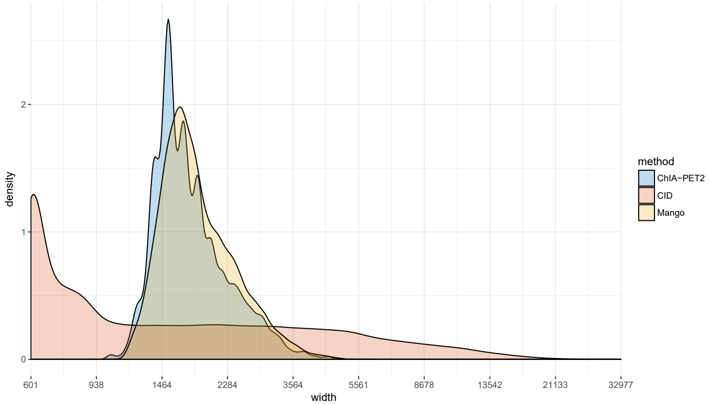

Snyder.GM19238.RAD21 (rep2) anchor width distribution

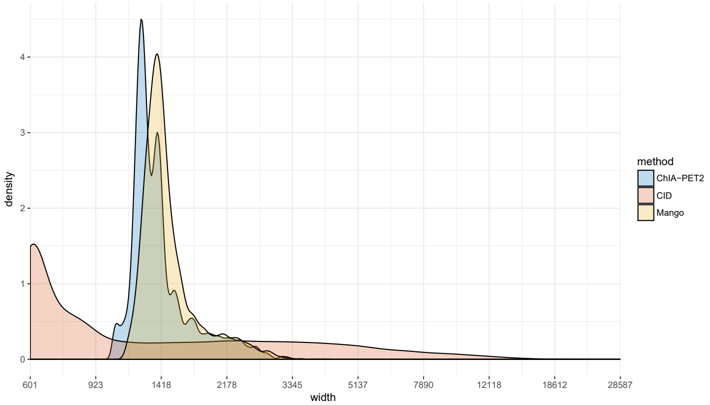

Snyder.GM19239.RAD21 (rep1) anchor width distribution

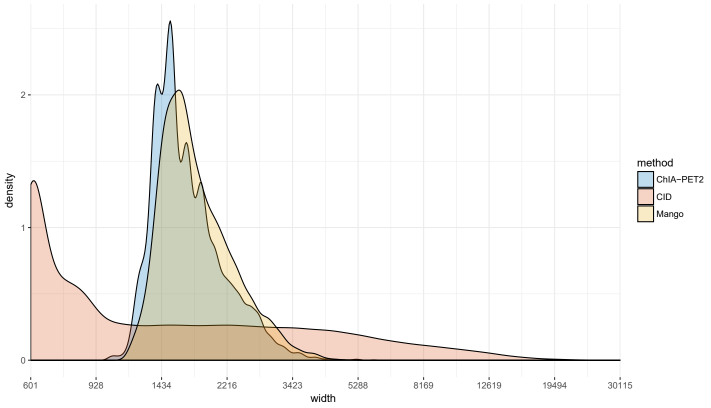

Snyder.GM19239.RAD21 (rep2) anchor width distribution

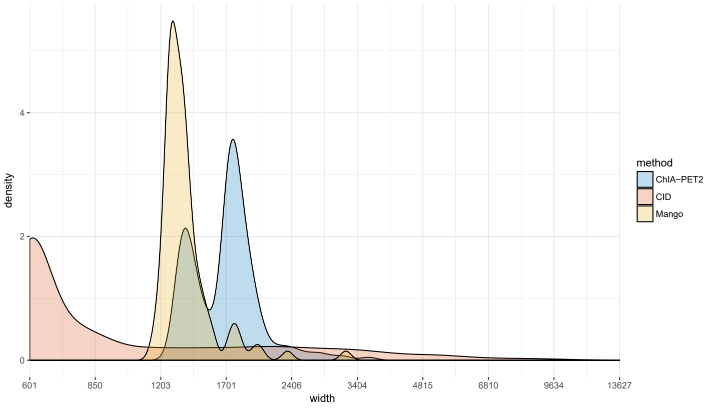

Snyder.GM19240.RAD21 (rep1) anchor width distribution

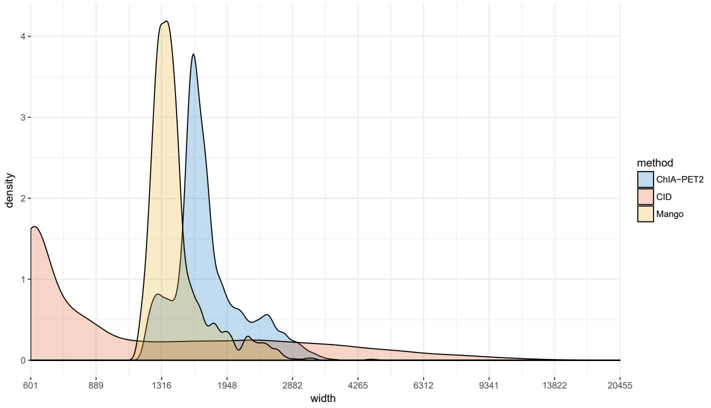

Snyder.GM19240.RAD21 (rep2) anchor width distribution

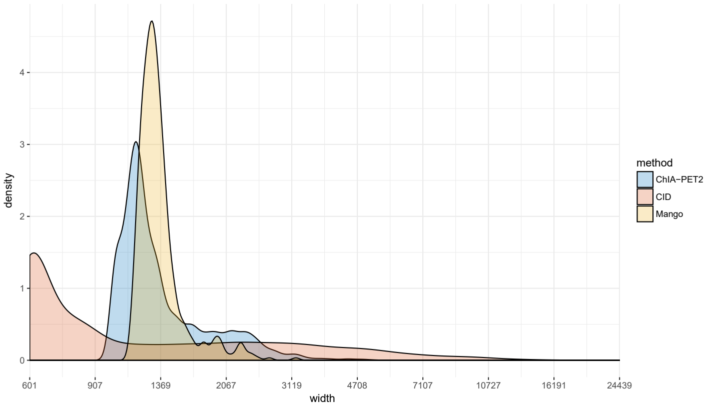

Snyder.HepG2.RAD21 (rep1) anchor width distribution

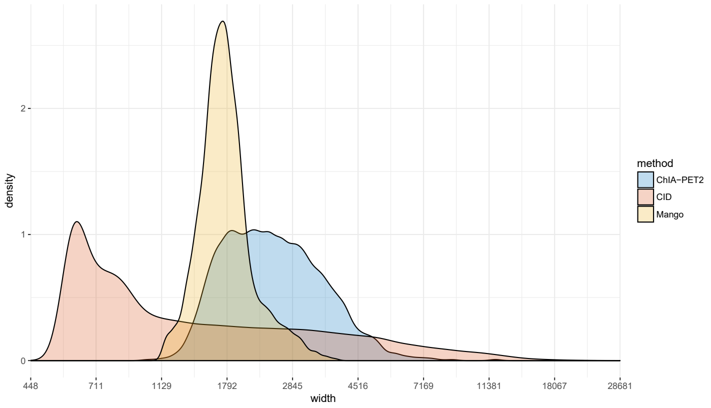

Snyder.HepG2.RAD21 (rep2) anchor width distribution

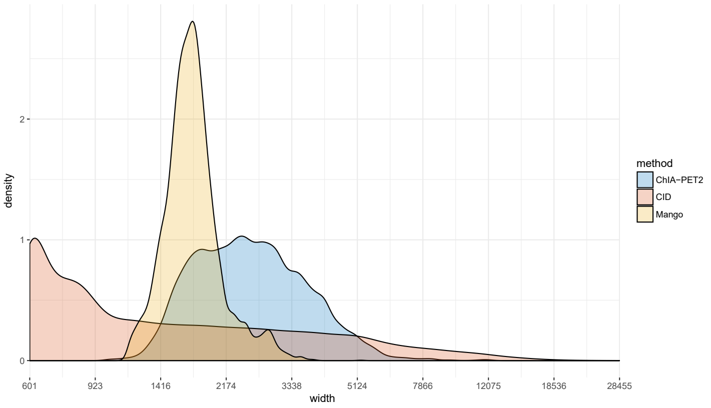

Snyder.JurkatCloneE61.RAD21 (rep1) anchor width distribution

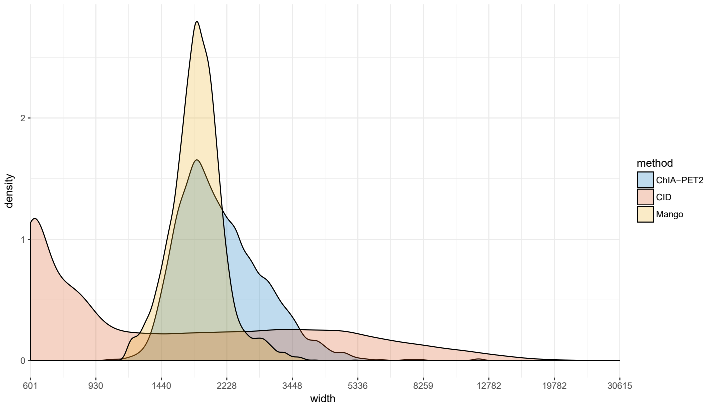

Snyder.JurkatCloneE61.RAD21 (rep2) anchor width distribution

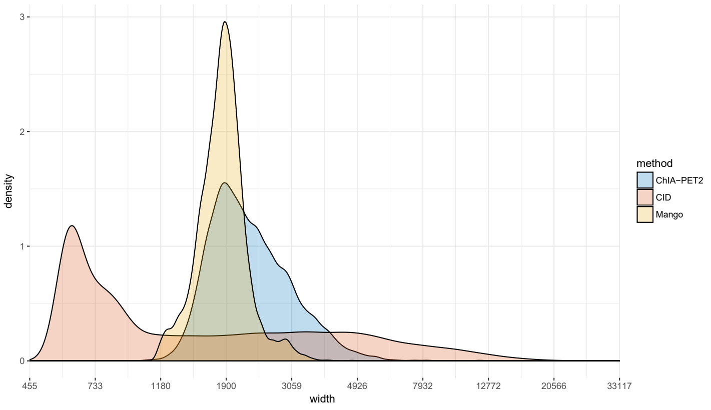

Snyder.K562.POLR2A (rep1) anchor width distribution

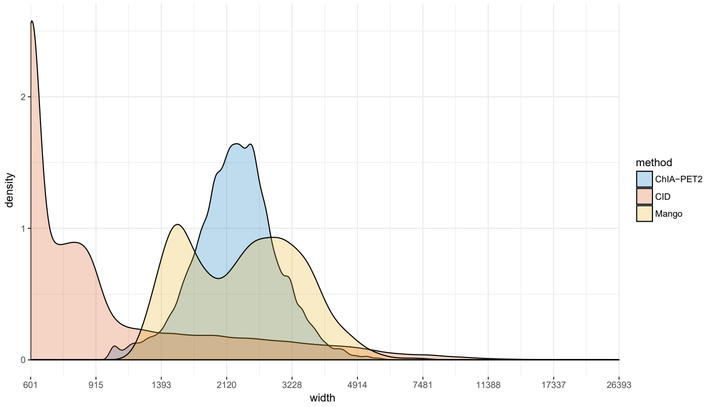

Snyder.K562.POLR2A (rep2) anchor width distribution

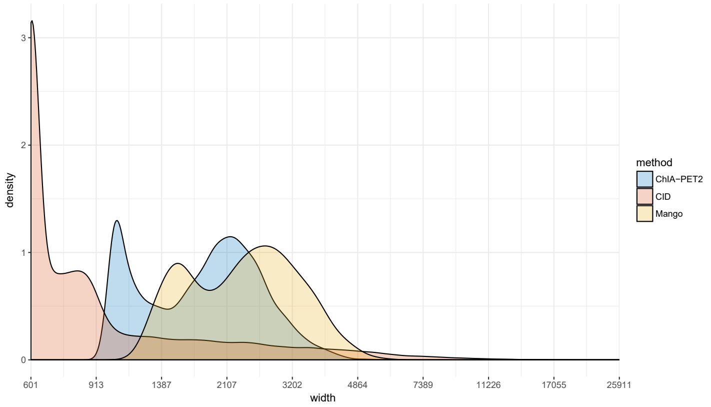

Snyder.K562.RAD21 (rep1) anchor width distribution

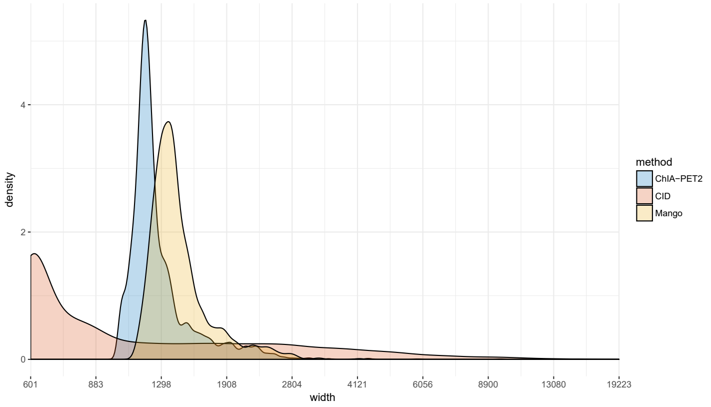

Snyder.K562.RAD21 (rep2) anchor width distribution

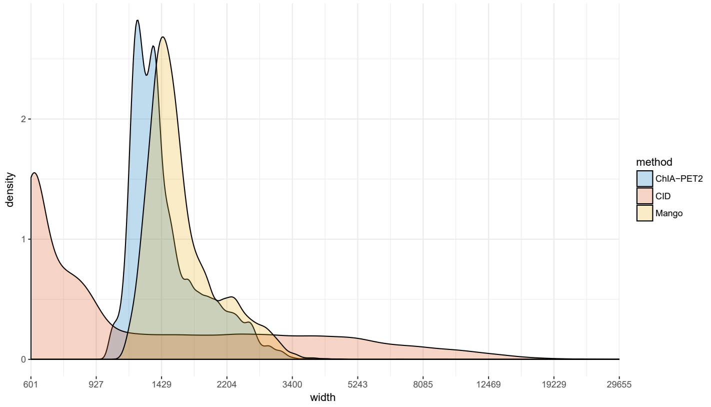

Snyder.LNCaPCloneFGC.RAD21 (rep1) anchor width distribution

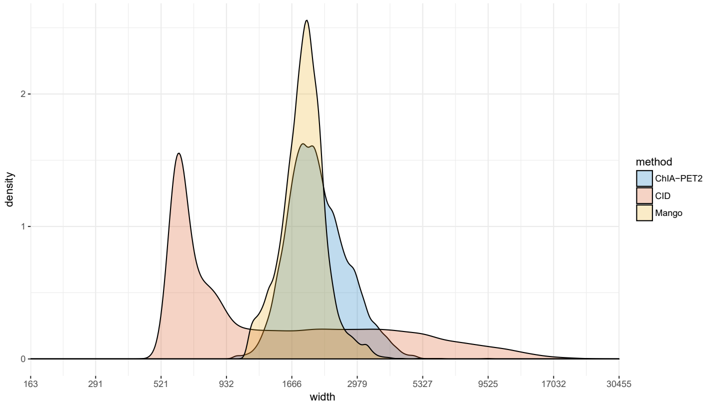

Snyder.LNCaPCloneFGC.RAD21 (rep2) anchor width distribution

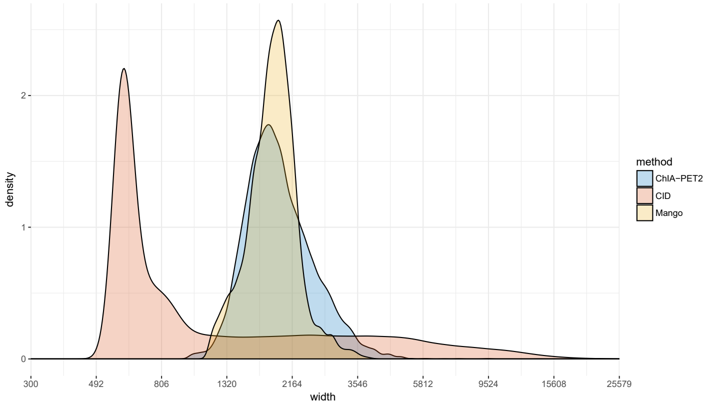

Snyder.MCF−7.RAD21 (rep1) anchor width distribution

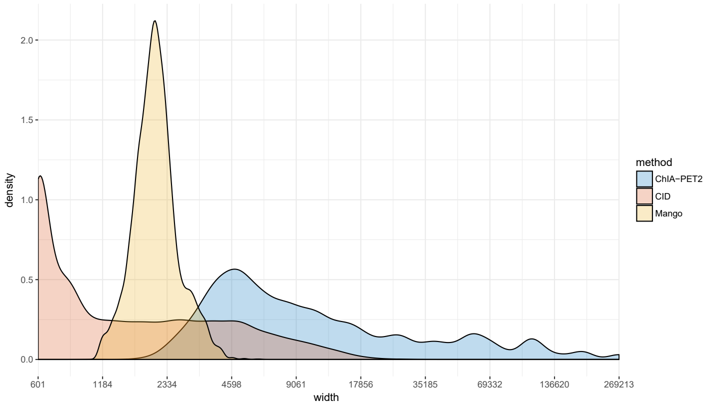

Snyder.MCF−7.RAD21 (rep2) anchor width distribution

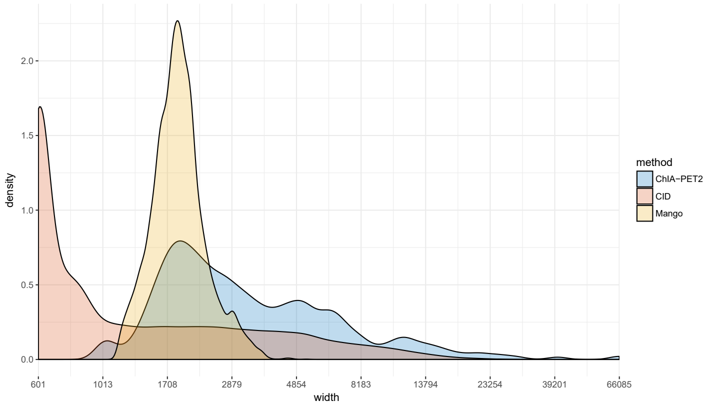

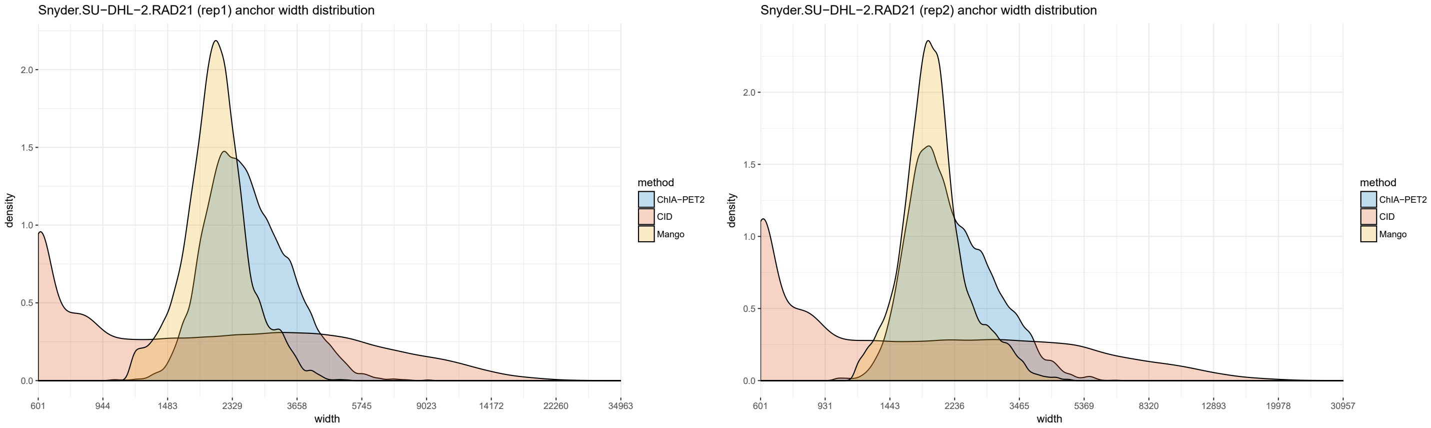

Figure S5: Comparing anchor width distributions of significant interactions identified by ChIA-PET2, CID, and Mango across 17 ENCODE data sets. Only anchors of intra-chromosomal interactions are considered.

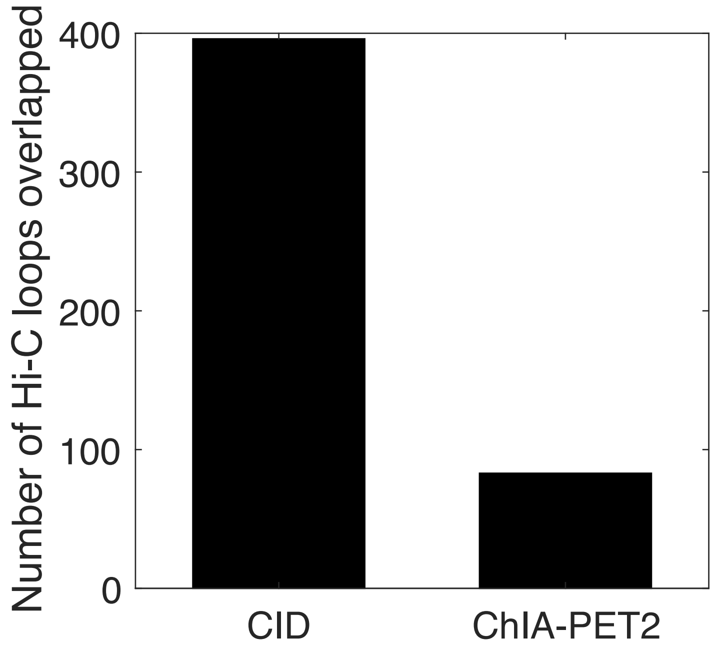

Figure S6. Number of Hi-C loops in K562 cells that overlapped with top 7498 interactions called by CID and ChIA-PET2 from POLR2A ChIA-PET data in K562 cells.

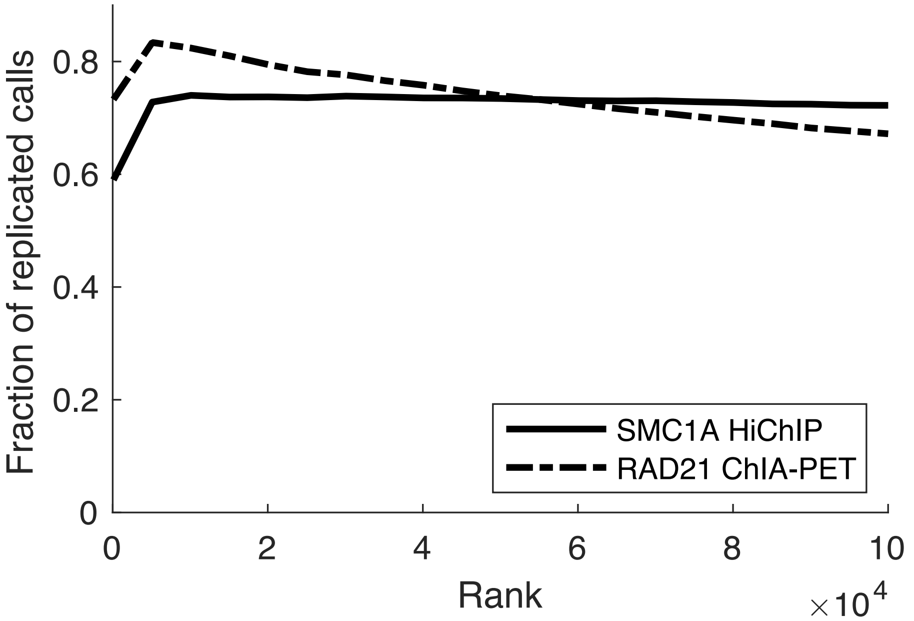

Figure S7. Interaction calls by CID from SMC1A HiChIP and RAD21 ChIA-PET data in GM12878 cells are similar in replicate consistency. Accumulative fractions of replicated interaction calls are computed using top ranking interactions at increasing ranks. Top 100,000 calls are shown.
